# Individual Consistency of Feather Pecking Behavior in Laying Hens: Once a Feather Pecker Always a Feather Pecker?

**DOI:** 10.3389/fvets.2015.00006

**Published:** 2015-04-14

**Authors:** Courtney L. Daigle, T. Bas Rodenburg, J. Elizabeth Bolhuis, Janice C. Swanson, Janice M. Siegford

**Affiliations:** ^1^Animal Behavior and Welfare Group, Department of Animal Science, Michigan State University, East Lansing, MI, USA; ^2^Behavioural Ecology Group, Wageningen Institute of Animal Sciences, Wageningen University, Wageningen, Netherlands; ^3^Adaptation Physiology Group, Wageningen Institute of Animal Sciences, Wageningen University, Wageningen, Netherlands

**Keywords:** feather pecking, laying hen, addiction, behavioral consistency, victim, neutral

## Abstract

The pecking behavior [severe feather, gentle feather, and aggressive pecks (AP)] of individual White Shaver non-cage laying hens (*n* = 300) was examined at 21, 24, 27, 32, and 37 weeks. Hens were housed in 30 groups of 10 hens each and on 3 cm litter with access to a feeder, perch, and two nest boxes. The number of severe feather pecks given (SFPG) and received (SFPR) was used to categorize hens as feather peckers (P), victims (V), neutrals (N), or feather pecker-victims (PV) at each age. Hens categorized as PV exhibited pecking behaviors similar to P and received pecks similar to V. SFP given were correlated with APs given, but not with gentle feather pecks (GFP) given throughout the study. State-transition plot maps illustrated that 22.5% of P remained P, while 44% of PV remained PV throughout the duration of the study. Lifetime behavioral categories identified hens as a consistent feather pecker (5%), consistent neutral (3.9%), consistent victim (7.9%), consistent feather pecker-victim (29.4%), or inconsistent (53.8%) in their behavioral patterns throughout their life. Consistent feather peckers performed more SFP than hens of other categories, and consistent neutral hens received fewer GFP than consistent feather PV. No differences in corticosterone or whole blood serotonin levels were observed among the categories. Approximately, half of the population was classified as a feather pecker at least once during the study, while the remainder was never categorized as a feather pecker. Therefore, even if the development and cause of feather pecking may be multifactorial, once the behavior has been developed, some hens may persist in feather pecking. However, as some hens were observed to never receive or perform SFP, emphasis should be made to select for these hens in future breeding practices.

## Introduction

Behavioral syndromes in nature are the result of natural selection for optimal survival in different environments (1). However, in agricultural animals, animal breeding, the animal’s environment, and social group compositions are regulated by humans. Therefore, the natural process of selection can no longer influence what behavioral characteristics are successful. As agricultural animals have been selected for high productivity and low input costs, we may have inadvertently selected, housed, or managed for individuals that perform unwanted behaviors that have severe social and environmental ramifications, such as the socially transmitted behavior of feather pecking in laying hens (2, 3).

One such unwanted behavior that may have been exacerbated by human selection and management practices is feather pecking. Feather pecking is a welfare concern for laying hens as well as an economic concern for the producer as this detrimental behavior can damage feather cover causing increased feed costs as well as result in injury and potentially cannibalism that is very difficult to control (4). This worldwide phenomenon is present in flocks of laying hens that differ in flock size (4), stocking density (5, 6), hen strain (7), or housing system (8). Substantial research has investigated the interaction between temperament and feather pecking in flocks of laying hens [for a review, see (9)], and once feather pecking has been observed in a flock of hens, this behavior is more likely to be observed in the same flock at a later age (10). However, little is known about whether individuals performing feather pecking are consistent in their pecking behavior throughout the lay cycle. Hens housed in large groups look phenotypically similar from a human perspective, which can make individual hen behavior challenging to measure. Yet, if the tendency to develop feather pecking is a manifestation of a behavioral syndrome, and if behavioral syndromes are consistent across time and context, then we would anticipate that hens performing feather pecking early in life would continue to do so later in life. Additionally, hens that do not engage in pecking behavior may consistently avoid these types of interactions – yet the validity of this assumption has not been verified.

Furthermore, although not specifically researched, different types of feather pecking behavior may stem from different motivations. Gentle feather pecking (GFP) has been observed during dust bathing (11), in low light levels (12), and could be interpreted as allopreening in some contexts. During normal GFP, the recipient does not usually react or move away. Severe feather pecking (SFP) can occur at the end of a GFP bout, or as a single event. The recipient usually moves away from the hen performing the SFP, reducing the amount of time the two hens spend in close proximity to one another. The singularity of SFP when compared to GFP suggests that the severe feather peck represents a release or satisfaction for the hen performing the pecking. Therefore, SFP may be a positive experience for the feather pecker and detrimental to the recipient, while GFP may be a neutral (or even potentially pleasurable) experience for both hens involved.

Aggressive pecking, normally directed at the head, functions to establish and maintain a social hierarchy, and can result in one hen acquiescing to another in competitive interactions (e.g., access to feeder or nest box). Because the end results from these types of pecking behaviors and social interactions are different, hens are expected to have different motivations for performing gentle or SFP or aggressive pecks (AP). Therefore, different mechanisms may be involved in the development and performance of these pecking behaviors.

An emerging theory within the feather pecking literature is that some hens may be consistently targeted as the victims of feather pecking. Victims have been characterized as having a homozygous wild-type allele at the PMEL17 locus resulting in more pigmented feathers compared to heterozygous or homozygous dominant individuals (13, 14), and higher rates of serotonin turnover in the dorsal thalamus, a region of the brain important for controlling compulsion compared to hens never engaged in feather pecking behavior (15). Bennewitz et al. (16) and colleagues in their examination of the heritability of pecking behavior illustrated that the giving of APs and feather pecks was heritable, while receipt of feather pecks was not heritable. These results align with findings from previous research investigating heritability of performing and receiving feather pecking (17) throughout the hens’ lifetime (18). Therefore, there may be parameters [e.g., UV visible feather patterns (19), pheromone release associated with hypothalamic expression profiles] not detectable via the sensory abilities of humans that identify individual hens who would be targets of feather pecking.

In flocks experiencing feather pecking, some hens have been observed to never deliver or receive feather pecks or APs. Their apathy toward conspecific pecking may be related to lower levels of serotonin turnover observed in the dorsal thalamus (e.g., higher impulse control), observed in non-peckers compared to feather pecking and victim counterparts (15). This suggests that hens that do not engage in either giving or receiving pecking behavior may have a different brain reactivity, and may be consistent in their lack of pecking behavior, throughout their lifetime. Brunberg et al. (20) compared brain gene expression in feather peckers, victims, and control birds and found that feather peckers were different from both victims and control birds, but victims and controls had similar expression profiles.

Possession of the characteristics associated with being a victim of feather pecking does not necessarily preclude a hen from having the motivation to perform feather pecking behavior, causing some hens to be caught on both sides of the proverbial fence. Thus, even though a hen may have the motivation to feather peck, she may also possess the characteristics that makes her a target of feather pecking. Alternatively, though all hens have the ability to both give and receive feather pecks, some may never feather peck yet will receive feather pecks, creating a “victim syndrome.” One factor that makes the “victim syndrome” challenging to quantify is that these victims may be culled due to injury or cannibalized by their peers. Therefore, identifying whether they consistently receive feather pecks is confounded by removal or mortality, which makes understanding their experience and subsequent welfare difficult but nonetheless important. Ultimately, four distinct behavioral phenotypes may exist within a flock of laying hens: (1) feather peckers (P) that engage in feather pecking and are never the recipient, (2) victims (V) that never perform feather pecking yet receive feather pecks, (3) non-feather pecker neutrals (N) that do not perform or receive feather pecks, and (4) feather pecker-victims (PV) that both receive and perform feather pecks.

Our objective was to identify whether individual hens were consistent in their pecking behavior throughout the duration of the study. Specifically, we hypothesized that individual feather pecking behavior (both gentle and severe, and giving and receiving pecks) would be consistent across a range of hen ages. Also, we hypothesized that hens performing GFP would be more likely to perform SFP at the same age. Hens receiving severe feather pecks (SFP) were expected to have higher levels of post-stress corticosterone levels than hens performing pecking without receiving any pecks (P) or hens not engaged in giving or receiving pecks (N). Hens performing feather pecks (P and PV) were expected to have lower concentrations of serotonin than hens in the other categories. We also hypothesized that aggressive pecking and feather pecking behavior would not be correlated across ages, as aggressive pecking is part of establishing a social hierarchy.

## Materials and Methods

### Animals and housing

All procedures were approved by the Michigan State University Institutional Animal Care and Use Committee (AUF 04/12-068-00). Thirty identical pens (1.5 m × 2.7 m) were constructed at the Michigan State University Poultry Teaching and Research Center. Pens were separated by floor to ceiling wire mesh, and temperature was regulated with forced heating and fan ventilation. Each pen was furnished with a commercial tube feeder, a water line containing three nipples, two nest boxes, and two wooden perches providing 1.5 m of available perch space. The floor was covered with 3 cm of litter (wood shavings). Litter depth was monitored weekly, and excess litter was removed when the depth surpassed 3 cm. Hens were exposed to incandescent lighting for 13.5 h/day (05:30–19:00 hours), which measured 21.1 ± 0.9 lux at hen level.

Each pen housed 10 White Shaver infrared beak-trimmed laying hens, which were randomly placed into pens at 16 weeks. Hens were beak trimmed as part of regular husbandry practices in the United States, and the hens were less likely to severely damage their flock-mates throughout the duration of the study. At placement, each hen was fitted with a plastic leg band to ensure that they were individually identifiable for the duration of the study, including when blood was collected and feathers were scored. At 18 weeks, the back of each hen was also colored with livestock marker in different color combinations to facilitate individual recognition on video recordings. Livestock marker (LA-CO^®^ Industries, Inc., Elk Grove Village, IL, USA) was reapplied prior to each video recording session. After manual restraint (MR) test completion (see Manual Restraint Test and Blood Sampling below), hens that were not tested were gently handled and had livestock marker reapplied. Hens are less likely to identify a social target for aggression if all hens are marked equally (21), which has been shown to be particularly important for hens housed in small groups (22).

### Treatments

When the hens were 22 weeks, three treatments (10 pens/trt) were randomly applied to the 30 pens as part of a separate experiment. The results of this experiment demonstrated that a hay bale in the hen’s environment has the potential to reduce the prevalence of GFP, but does not impact levels of SFP (23). The three treatments of this experiment were as follows. (1) HAY – one 5 kg hay bale consisting of approximately three flakes of hay held together with twine and measuring approximately 0.38 m long × 0.38 m wide × 0.38 m tall. (2) BOX – a clear plastic box (Rubbermaid^®^ Roughneck^®^ Clear 17.9 L, Rubbermaid, High Point, NC, USA) measuring 0.42 m long × 0.27 m wide × 0.27 m tall, filled with loose hay that was visible to hens through the clear sides and bottom of the box and weighted with a cinderblock to ensure that the hens did not turn the box over. One box was placed upside down in the litter of each BOX pen so the lid was not accessible to the hens. (3) CON – a negative control where no treatment was applied to the pen.

Hay bales were checked bi-weekly to ensure that they remained intact. As needed, loose hay was removed from a pen and replaced with a new bale. Partially destroyed bales were assessed, and replaced as necessary on a case-by-case basis. Each HAY pen had the hay bale replaced a minimum of three times throughout the duration of the study. At 24 weeks, a gap was found in the wire separating two adjoining HAY and BOX pens, and the hens were observed moving between the two pens. Therefore, both pens were removed from the study.

### Manual restraint test and blood sampling

At 21, 24, 27, 32, and 37 weeks, 120 hens were randomly selected, balanced across treatments and pen location within the barn, and subjected to a MR. Birds were tested in random order and were not taken from the same or neighboring pens in consecutive tests. The sampled hens selected were balanced across trials so that each hen was selected a minimum of two times and a maximum of three times throughout the duration of the study. The MR procedure used in this study has been previously described in Uitdehaag et al. (24). Briefly, a bird was taken out of its home pen and placed on its side on a flat surface for 5 min in a quiet room adjacent to the room where its home pen was located. The tester used one hand to loosely restrain the bird’s legs, while the other hand was placed over the upper part of the bird’s body. The hand restraining the legs mainly functioned to prevent the bird from escaping if it struggled or righted itself, whereas the mild pressure from the other hand encouraged the hen to remain recumbent. After any struggle, birds were gently returned to their original position. Parameters measured during the manual restraint included latency to struggle, latency to vocalize, number of vocalizations, and number of struggles. Each MR was performed by one of two persons on two consecutive days between 9:00 and 14:00 hours.

After the MR, each hen was placed individually in a plastic transportation crate located in a hallway adjacent to the testing room. The hen remained in the transportation crate until 15 min had elapsed following removal from her home pen, to allow the corticosterone response to reach its peak (25). Then, a 2.5 mL blood sample was taken from a brachial vein using a 22-guage needle and 3-mL syringe. Prior to blood collection, the needle was flushed with 0.9% NaCl concentrated with EDTA to prevent clotting in the needle and syringe. A portion of the blood sample (~1 mL) was separated and stored at −80°C until serotonin (5-HT) analysis. The remainder was immediately centrifuged to separate phases for corticosterone analysis.

After blood collection, the feather condition of multiple body parts (head, neck, back, rump, underneck, coverts, breast, legs, belly, wing-primary feathers, and tail feathers) was scored on a 0–5 scoring system as described in Bilcik and Keeling (26). A high score represented poor feather condition/cover while a low score represented good feather condition/cover. After feather scoring, the hens were returned to her home pen. Feather scores from all body parts were summed to provide a whole body score.

### Serotonin analysis

Most serotonin (5-HT) in avian blood is localized in platelets (27), and 5-HT concentrations in whole blood have been shown to correlate (*r* = 0.34–0.57) with brain 5-HT concentrations (28). Therefore, we analyzed whole blood samples following a previously validated protocol (29). Briefly, 5-HT levels in whole blood (1 mL) were determined by a fluorescence assay. Whole blood was pipetted into 50 mL centrifuge tubes to which was added 2 mL of 0.9% NaCl solution, 1 mL of an ascorbic acid solution (3% in deionized water saturated with KCl and EDTA), and 5 mL of a phosphate buffer (2 M K_2_HPO4, saturated with KCl and adjusted to pH 10 with KOH), followed by 20 mL of *n*-butanol. Tubes were shaken thoroughly for 5 min and centrifuged (Allegra X-15R, Beckman Coulter Inc., Indianapolis, IN, USA) at 895 g for 15 min. Fifteen milliliters of the butanol layer was transferred to a second tube containing 2 mL of 0.1 M HCl and 25 mL of cyclohexane, and tubes were shaken for 20 s then centrifuged for 4 min at 895 g. The butanol-cyclohexane layer was removed, and 1 mL of the acidic phase was pipetted in a tube containing 0.3 mL of 12 M HCl that was then vortexed for 3 s. Samples were pipetted in triplicate at a volume of 250 μl into a 96-well plate, and fluorescence was determined using a fluorometer (SpectraMax Gemini EM, Molecular Devices, Sunnyvale, CA, USA) set at an excitation of 295 nm and an emission of 540 nm. A standard curve was prepared by taking 0.1–0.5 mL of serotonin hydrochloride (Sigma-Aldrich) dissolved in Krebs–Ringer-phosphate buffer (0.2755 μmol/mL) then diluted to a volume of 1 mL with 0.9% NaCl solution. Each dilution was subjected to the procedure as described above.

### Corticosterone analysis

Immediately after blood collection, blood samples were centrifuged at 930 *g* for 6 min at 4°C. Plasma was transferred to a 1.7 mL mini-tube with a transfer pipette and stored at −80°C until analysis. Hormone measurements were carried out according to manufacturer instructions in triplicate for each sample using a micro plate enzyme-immunoassay (Cayman Chemical, Grand Rapids, MI, USA). All samples were diluted to 1:3 with assay dilutant prior to analysis.

### Behavioral observations in the home pen

Twenty-four hours prior to each MR (at 21, 24, 27, 32, and 37 weeks), ceiling-mounted video cameras (VF-540 Bullet Camera, Clinton Electronics Corp., Loves Park, IL, USA) recorded (at 30 frames/s) hen behavior during two 30-min periods (7:30–8:00 hours and 15:30–16:00 hours) during the light period, similar to the recording protocol used by Rodenburg and Koene (30). The number of pecks each hen gave to the enrichment (EP; BOX and HAY only), the number of AP, and SFP and GFP were recorded. Further, these counts of pecks were identified as the number of aggressive pecks given (APG) and received (APR), the number of severe feather pecks given (SFPG) and received (SFPR) as well as the number of gentle feather pecks given (GFPG) and received (GFPR) (Table 1).

**Table 1:** **Description of pecking behaviors observed in the home pen**.

Pecking behavior	Description
Gentle feather pecking (GFP)	Hen uses beak to gently peck at feathers of conspecific. This pecking is normally ignored by the recipient and usually does not result in the removal of a feather. Usually occurs in bouts where the hens will GFP several times in a single bout. Normally directed at the back or tail, but may be directed at the head. Count total number of pecks
Severe feather pecking (SFP)	Hen uses beak to forcefully peck at victim. Victim will usually respond to pecking by moving away or retaliating. May result in removal of a feather. Usually occurs as a single event, but may happen twice in a row. Will not occur in bouts. Usually directed toward the back, rump, or tail, but may be directed at the head. Count total number of pecks
Aggressive pecking (AP)	Occurs when one hen raises her head and forcefully stabs beak either once or multiple times at another hen. Aggressive pecks will usually be directed at the head, but may also be directed at the body. The recipient will usually show avoidance behavior by ducking or moving away from aggressive bird. May be associated with a chase, standoff, or leap. Count total number of pecks
Enrichment pecking (EP)	Hen uses beak to peck at top or sides of hay bale or plastic box (HAY and BOX rooms only). Count total number of pecks

### Statistical analysis

All analyses were performed using SAS 9.4 (SAS Institute Inc., Cary, SC, USA). Each response parameter was tested for normality and heterogeneity of variance prior to analysis, and the number of AP, SFP, and GFPG and GFPR, as well as feather scores was log transformed to meet assumptions of normality. Previous research investigating the impact of the environmental enrichment treatments on pecking behavior identified that a larger number of GFP given were performed in the CON treatment compared to the HAY and BOX treatments (23). Therefore, to account for the possible impact of treatment, each response parameter was analyzed separately for the effect of treatment using a Generalized Linear Mixed Model (PROC MIXED). The model included the fixed effect of treatment. The residuals from each analysis were saved and used for subsequent analyses. A correlation on these residuals was conducted (PROC CORR) to identify the associations among pecking behavior at all ages. Significance was determined as *P* < 0.05.

Further, each individual was placed into one of four possible categories based upon its individual pecking behavior at each age and descriptive statistics were calculated. Hens were categorized as feather peckers (P), victims (V), neutrals (N), or feather pecker-victims (PV) at each age. Hens were categorized based upon whether they gave or received any SFP (Table 2).

**Table 2:** **The number (percentage) of hens per category across time, the criteria used to assign individuals hens into one of four different categories based upon the number of severe feather pecks (SFP) each hen gave and received**.

Category	Criteria	Count (percentage) of hens				
		21 weeks	24 weeks	27 weeks	32 weeks	37 weeks
Feather pecker	Receive 0 SFP	26 (14.4)	35 (17.6)	41 (17.8)	47 (21.6)	29 (15.3)
	Give > 1 SFP	
Neutral	Receive 0 SFP	23 (12.8)	61 (30.7)	32 (13.9)	41 (18.8)	27 (14.2)
	Give 0 SFP	
Victim	Receive >1 SFP	26 (14.4)	42 (21.1)	49 (21.3)	61 (28.0)	29 (15.3)
	Give 0 SFP	
Feather pecker-victim	Receive >1 SFP	105 (58.3)	61 (30.7)	108 (47.0)	69 (31.7)	105 (55.3)
	Give >1 SFP	

Using this categorical data set, analyses were conducted to determine whether an individual remained in the same behavioral category throughout the duration of the study. To visually analyze the probability that a hen would stay in the same state later in life, state-transition matrices were calculated. These matrices illustrate the probability that once a hen was placed in a category that it would either remain in the same category, or would be placed in a different category at the next data collection time point. Transition matrices were calculated in R 3.1.1 (R Foundation for Statistical Computing, Vienna, Austria) and plot maps were constructed with the diagram package.

Finally, the data were analyzed to identify how consistently hens were placed into the same behavioral category (P, N, V, PV) throughout their lifetime. Since hen behavior was used to categorize individual hens at five different ages, hens that received a “feather pecker (P)” categorization three out of the five ages observed were labeled “consistent feather peckers (CP).” Hens that received a “neutral” categorization three out of the five observed ages were labeled “consistent neutrals (CN).” The same process was repeated for “victims” and “feather pecker-victims” resulting in hens that were classified as “consistent victims (CV)” and “consistent feather pecker-victims (CPV).” Some hens did not fall into a single category at least three out of the five times, and were subsequently labeled as “inconsistent (IC).” A Generalized Linear Mixed Model (PROC MIXED) on the residuals was utilized (described above) to identify whether there were differences among the five consistency categories for the number of vocalizations, number of struggles, latency to struggle, latency to vocalize, the number of SFP, GFP, and APG and APR, as well as the concentration of whole blood serotonin and corticosterone. Differences among the categories were identified using Least Squared Means with a Tukey–Kramer adjustment.

## Results

### Descriptive statistics

**Figure 1:**
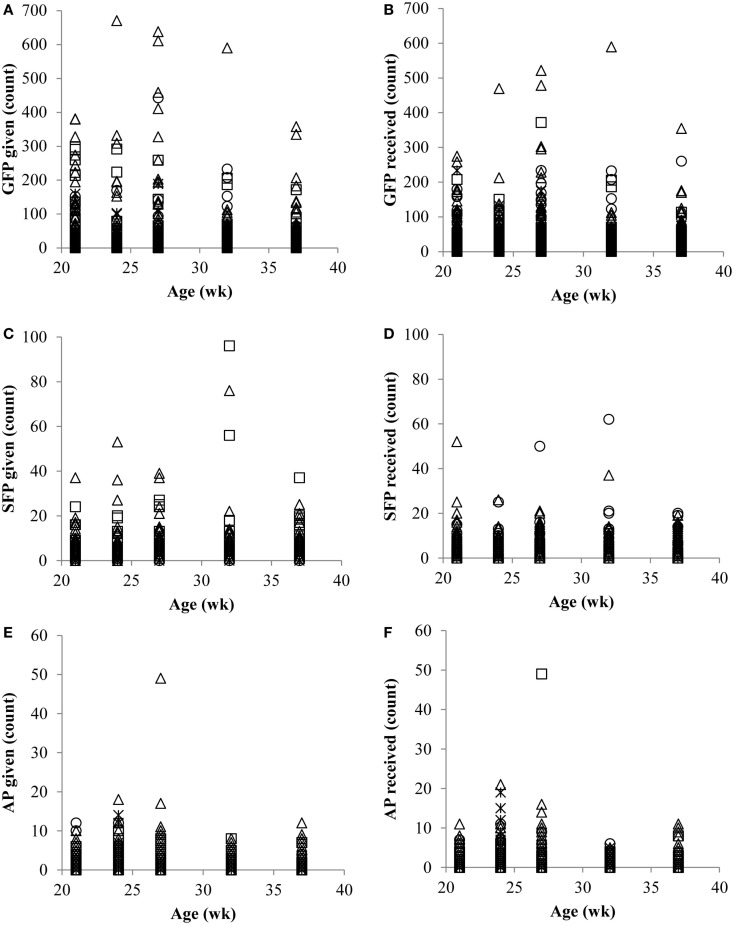
Number (counts) of gentle feather pecks (GFP) given (A) and received (B), number of severe feather pecks (SFP) given (C) and received (D), and number of aggressive pecks (AP) given (E) and received (F) by Neutrals (*), Feather Peckers (□), Pecker-victims (Δ), and Victims (∘).

Across all ages, on average, the largest percentage (44.6) of observed hens was categorized as feather PV. Feather peckers (P) represented the smallest proportion of the observed hens (17.3), while neutrals (N; 18.1) and victims (V; 20.0) composed the remainder. Counts (and percentages) of hens within each category across all ages are listed in Table 2. The number of GFP given (Figure 1A), GFP received (Figure 1B), SFP given (Figure 1C), SFP received (Figure 1D), AP given (Figure 1E), and AP received (Figure 1F) at each age point are presented in Figure 1.

Feather scores increased as the hens aged (*F*_4,373_ = 142.67, *P* < 0.0001), but did not differ among the four categories at each age (Table 3) illustrating that feather damage became more severe with hen age.

**Table 3:** **The number (mean ± SEM) of gentle feather pecks given (GFPG), gentle feather pecks received (GFPR), severe feather pecks given (SFPG), severe feather pecks received (SFPR), aggressive pecks given (APG), and aggressive pecks received (APR) by individual hens in four behavioral categories [feather pecker (P), victim (V), neutral (N), and pecker-victim (PV)], at five different ages throughout the lay cycle**.

Age (weeks)	P	V	N	PV	*df*	*F*-value
**SFPG**
21	4.7 ± 1.1^a^	0	0	5.3 ± 0.5^b^	1,129	4.07*
24	3.9 ± 0.8	0	0	5.5 ± 1.1	1,94	0.69
27	5.8 ± 1.5	0	0	5.0 ± 0.6	1,147	0.09
32	7.4 ± 2.2	0	0	4.8 ± 1.1	1,114	1.25
37	4.4 ± 0.8	0	0	4.5 ± 0.4	1,132	0.04
**SFPR**
21	0	3.7 ± 0.4	0	5.1 ± 0.7	1,145	1.51
24	0	5.0 ± 0.7	0	4.6 ± 0.6	1,101	1.26
27	0	3.9 ± 1.0^a^	0	5.4 ± 0.4^b^	1,155	14.18***
32	0	5.9 ± 1.1	0	4.5 ± 0.6	1,128	1.14
37	0	4.5 ± 0.9	0	4.6 ± 0.4	1,131	0.51
**GFPG**
21	44.6 ± 14.1^a,b^	19.2 ± 4.0^a^	20.0 ± 6.6^a,b^	52.6 ± 7.8^b^	3,183	4.02**
24	29.0 ± 10.3	13.3 ± 4.3	11.1 ± 2.6	49.3 ± 13.6	3,143	2.59
27	23.0 ± 7.1^a^	25.8 ± 9.4^a^	19.4 ± 6.7^a^	53.9 ± 10.5^b^	3,194	5.73**
32	37.8 ± 19.2^a,b^	9.8 ± 1.6^a^	6.3 ± 1.5^a,b^	32.9 ± 7.1^b^	3,170	3.13*
37	25.1 ± 6.4^a^	8.7 ± 2.5^b^	8.7 ± 3.0^a,b^	35.9 ± 5.6^a^	3,159	5.95**
**GFPR**
21	18.8 ± 6.3^a^	41.1 ± 6.4^b,c^	27.2 ± 8.5^a,c^	47.1 ± 5.3^b^	3,195	6.78***
24	31.4 ± 6.4	20.8 ± 4.6	13.80 ± 2.95	37.44 ± 8.82	3,166	2.11
27	23.8 ± 9.1^a,b^	32.4 ± 7.7^a,b^	17.31 ± 5.77^a^	51.26 ± 8.03^b^	3,209	3.5*
32	18.4 ± 5.9	25.7 ± 5.8	9.32 ± 2.33	30.19 ± 8.83	3,172	1.72
37	12.8 ± 4.3^a^	33.3 ± 9.4^a,b^	11.26 ± 3.26^a^	32.35 ± 4.38^b^	3,163	7.97***
**APG**
21	1.1 ± 0.3	0.8 ± 0.3	0.24 ± 0.13	1.5 ± 0.2	3,87	0.8
24	1.69 ± 0.41	1.07 ± 0.36	2.11 ± 0.44	2.67 ± 0.43	3,103	1.14
27	1.58 ± 0.31^a^	0.43 ± 0.12^a^	0.41 ± 0.12^a^	3.37 ± 0.52^b^	3,125	10.18***
32	1.66 ± 0.27^a^	0.44 ± 0.10^b^	0.49 ± 0.12^a,b^	1.43 ± 0.22^a,b^	3,103	2.82*
37	0.89 ± 0.26	0.79 ± 0.22	0.30 ± 0.13	2.36 ± 0.24	3,108	3.27*
**APR**
21	0.8 ± 0.2	1.3 ± 0.3	0.24 ± 0.1	1.6 ± 0.2	3,98	3.65*
24	1.00 ± 0.28^a^	1.98 ± 0.35^a,b^	2.48 ± 0.49^b^	2.82 ± 0.47^a,b^	3,121	3.64*
27	1.49 ± 1.19^a,b^	1.29 ± 0.27^a,b^	0.31 ± 0.14^a^	3.04 ± 0.32^b^	3,122	3.64*
32	0.66 ± 0.15	1.16 ± 0.21	0.76 ± 0.19	1.28 ± 0.18	3,98	1.35
37	0.86 ± 0.31^a,b^	1.32 ± 0.23^a,b^	0.30 ± 0.09^a^	2.18 ± 0.25^b^	3,104	4.97**
**Feather score**
21	1.0 ± 0.27	1.31 ± 0.21	1.33 ± 0.24	1.23 ± 0.12	3,68	0.49
24	1.67 ± 0.29	1.92 ± 0.26	1.90 ± 0.19	1.75 ± 0.18	3,57	0.21
27	2.50 ± 0.23	2.12 ± 0.21	2.67 ± 0.30	2.02 ± 0.17	3,89	1.46
32	4.55 ± 0.36	4.65 ± 0.37	3.77 ± 0.17	4.44 ± 0.33	3,84	0.65
37	7.13 ± 0.69	6.92 ± 0.70	8.80 ± 1.10	6.33 ± 0.31	3,75	2.40

*P < 0.05, **P < 0.01, ***P < 0.0001. Least squared means with a Tukey–Kramer adjustment were utilized to identify differences among the categories. Categories lacking a common superscript letter differ significantly (*P* < 0.05). SFPG was only compared between P and PV (V and N showed no SFPG, and as all values were 0 and had no variance these could not be included in the analysis), and SFPR was only compared between V and PV (P and N showed no SFPR, and again as all values were 0 and had no variance these could not be included in the analysis).

### Behavioral consistency

The number of SFPG did not differ between P and PV at all ages except at 21 weeks where PV gave more SFP than P, and the number of SFPR did not differ between V and PV at all ages except 27 weeks (Table 3) where PV received more SFP than V. Irrespective of age, PV generally performed more GFP throughout the duration of the study compared to the other behavioral categories while receiving a level of GFP comparable to what was observed for V. Differences were observed among the four categories for the number of AP given at 27, 32, and 37 weeks with most AP being performed by PV, and more AP were received by PV at 21, 24, 27, and 37 weeks compared to the other categories.

**Figure 2:**
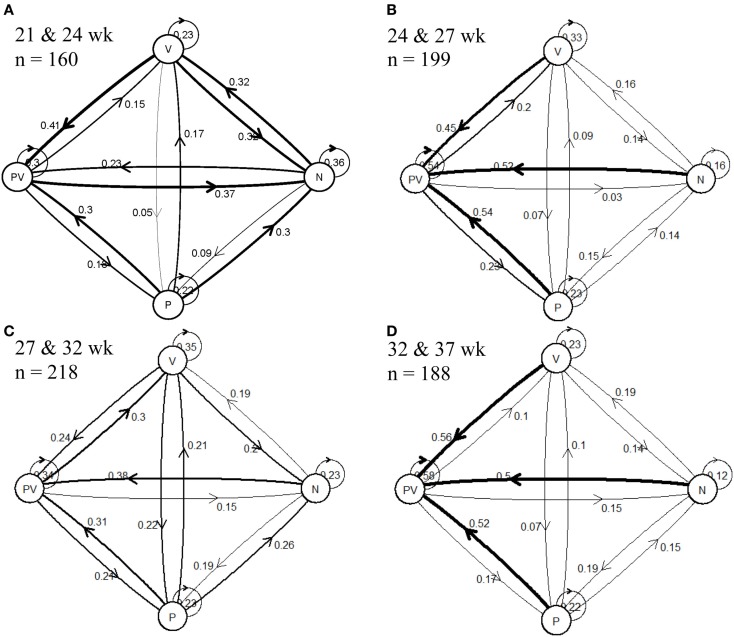
**State-transition plot maps for hen pecking categories [feather pecker (P), victim (V), neutral (N), and pecker-victims (PV)] between two consecutive ages at (A) 21 and 24 weeks, (B) 24 and 27 weeks, (C) 27 and 32 weeks, and (D) 32 and 37 weeks**. The numbers represent the proportion of the population that performed the transition indicated by the direction arrows.

As represented in the transitional matrices, of the hens classified as P, on average, 22.5% of hens were also categorized as P at the following time point (Figure 2), and this trend was consistent throughout the duration of the study. Victims were observed to remain V, on average, 28.5% of the time; 21.8% of N remained N; and 44% of PV remained PV. When hens changed categories between time points, the least prevalent transition was from V to P, where, on average, 10.3% of V hens became P at the following time point, while becoming a PV from being a P was the most prevalent (41.8%) transition.

The proportion of hens transitioning from N to P increased throughout the study from 9% between 21 and 24 weeks to 19% between 32 and 37 weeks. The proportion of hens transitioning from N to V was highest (32%) between 21 and 24 weeks, and remained relatively low (<19%) throughout the remainder of the study. A similar pattern was observed for hens transitioning from V to N and between PV and N, where the largest percentages were seen between 21 and 24 weeks. The transition to a PV was the most common behavioral transition observed. On average, 40.8% of N transitioned to PV, 41.5% of V transition to PV, and 41.8% of P transitioned to PV.

### Lifetime behavioral category analysis

Over the duration of the study, about half (53.8%) of the hens in this study had inconsistent behavioral profiles, while the remaining hens remained in their original behavioral category throughout the duration of the study. Further, 52% of hens were never categorized as a P at any point, while the remainder was classified as a P during at least one time period during the study. Hens that were placed into consistent behavioral categories were most often observed (29.4%) to be consistent feather PV (Table 4). The smallest proportion of the population was observed to be CN (3.9%), followed by CP (5.0%), and then CV (7.9%).

**Table 4:** **The number (percentage) of hens per each lifetime category [consistent neutral (CN), consistent feather pecker (CP), consistent pecker-victim (CPV), consistent victim (CV), and inconsistent (I)] along with the concentrations (mean ± SEM) of whole blood serotonin (5-HT, nanomoles per milliliter), plasma corticosterone (CORT, picogram per milliliter), as well as the number of gentle feather pecks given (GFPG), gentle feather pecks received (GFPR), severe feather pecks given (SFPG), severe feather pecks received (SFPR), aggressive pecks given (APG), aggressive pecks received (APR), and enrichment pecks (EP) performed by individual hens in five lifetime behavioral categories**.

	CN	CP	CPV	CV	CI	*df*	*F*-value
*n* (%)	11 (3.9)	14 (5.0)	82 (29.4)	22 (7.9)	150 (53.8)	–	–
5-HT	49.84 ± 3.46	51.62 ± 2.28	51.42 ± 1.5	49.14 ± 2.3	52.42 ± 0.92	4,232	0.56
CORT	1077.49 ± 150.67	1072.46 ± 97.17	908.11 ± 49.22	919.22 ± 84.79	1074.62 ± 48.9	4,232	1.74
SFPG	1.02 ± 0.47^a^	8.34 ± 2.52^b^	3.74 ± 0.34^a^	0.65 ± 0.13^a^	2.3 ± 0.27^a^	4,179	3.62*
SFPR	1.4 ± 0.23	0.81 ± 0.16	3.97 ± 0.27	4.73 ± 0.63	2.3 ± 0.23	4,180	0.72
GFPG	25.61 ± 10.49	40.25 ± 20.34	32.69 ± 4.83	9.74 ± 1.26	30.61 ± 6	4,187	1.90
GFPR	12.15 ± 2.86^a^	14.88 ± 3.44^a,b^	31.24 ± 3.9^b^	32.73 ± 6.88^a,b^	31.31 ± 4.02^a,b^	4,187	2.79*
APG	0.58 ± 0.17	2.22 ± 0.53	2.11 ± 0.18	1.29 ± 0.27	1.3 ± 0.13	4,167	0.43
APR	1.02 ± 0.38	1.05 ± 0.17	2.11 ± 0.22	2.04 ± 0.29	1.32 ± 0.16	4,170	1.98
EP	5.5 ± 1.57	2.15 ± 0.9	3.89 ± 0.99	3.46 ± 1.21	3.67 ± 0.69	4,95	0.61

*Least squared means with a Tukey–Kramer adjustment were utilized to identify differences among the categories. Least squared mean differences (*P* < 0.05) among categories are represented by superscript lowercase letters*.

No differences were observed among the consistency categories for the number of struggles (*F*_4,190_ = 1.29, *P* = 0.27), the number of vocalizations (*F*_4,203_ = 0.45, *P* = 0.77), the latency to struggle (*F*_4,236_ = 1.87, *P* = 0.12), and the latency to vocalize (*F*_4,236_ = 0.10, *P* = 0.98) during a MR test.

The number of SFPG differed among the five lifetime categories (*F*_4,179_ = 3.62, *P* = 0.007). CP gave more SFP than CN (*t*_179_ = −2.92, *P* = 0.032), CPV (*t*_179_ = 3.47, *P* = 0.006), CV (*t*_179_ = 3.19, *P* = 0.014), and IC hens (*t*_179_ = 3.58, *P* = 0.004). No differences were observed among the five categories for the number of SFPR (*F*_4,180_ = 0.72, *P* = 0.58). Further, no differences were observed among the five lifelong categories for the number of GFPG (*F*_4,187_ = 1.90, *P* = 0.11); however, the number of GFPR differed (*F*_4,187_ = 2.79, *P* = 0.03) where CN received fewer GFP compared to CPV (*t*_187_ = −2.77, *P* = 0.048). The numbers of APG (*F*_4,167_ = 0.43, *P* = 0.78) and APR (*F*_4,170_ = 1.98, *P* = 0.01) did not differ among the five lifelong categories.

### Associations among different types of pecking behavior

The number of GFPG by an individual hen was positively correlated with the number of SFPG by that hen at the same age (Table 5). The number of GFPR by an individual hen was also positively correlated with the number of SFPR by that same hen. Unexpectedly, the number of SFPG was also correlated with the number of APG, and this relationship became stronger as the hens aged. The number of GFPG was not correlated with the number of APG.

**Table 5:** **Pearson’s correlation coefficients (*r*), with a Bonferonni correction, among the number of gentle feather pecks given (GFPG), gentle feather pecks received (GFPR), severe feather pecks given (SFPG), severe feather pecks received (SFPR), aggressive pecks given (APG), and aggressive pecks received (APR) by individual hens at five different ages throughout the lay cycle**.

Variable 1	Variable 2		Age (weeks)				
			21	24	27	32	37
GFPG	GFPR	*r*	0.09	0.057	0.162	0.091	0.107
		*n*	180	126	188	148	149
GFPG	SFPG	*r*	0.415*	0.413*	0.538*	0.488*	0.422*
		*n*	123	78	135	97	128
GFPG	APG	*r*	−0.029	−0.012	0.294*	0.196	0.05
		*n*	84	85	117	86	108
GFPR	SFPR	*r*	0.393*	0.436*	0.437*	0.408*	0.499*
		*n*	143	93	152	113	127
GFPR	APR	*r*	0.191	0.083	0.156	0.023	0.083
		*n*	101	108	122	83	104
SFPG	APG	*r*	0.068	0.282	0.279	0.356*	0.403*
		*n*	71	63	105	75	94
SFPR	APR	*r*	0.005	0.310	0.276	0.273	−0.002
		*n*	79	77	109	68	89
APG	APR	*r*	0.313	−0.024	0.209	0.274	−0.072
		*n*	52	74	79	51	69

Significance (*P* < 0.0001) is indicated by an * and bold numbers.

### Physiological responses

Corticosterone levels did not differ among the four behavioral categories (P, V, N, PV) at 21, 24, 27, 32, or 37 weeks (*P* > 0.05). However, differences in serotonin levels were observed at 21 weeks (*F*_3,66_ = 2.85, *P* = 0.04) where N hens had higher whole blood serotonin concentrations than V (*t*_69_ = −2.40, *P* = 0.02) or PV (*t*_69_ = −2.66, *P* = 0.01). No differences were observed among the four behavioral categories for whole blood serotonin concentration at later time points (24, 27, 32, and 37 weeks; *P* > 0.05).

No differences were observed among the five lifelong categories (CP, CN, CV, CPV, CI) for the concentration of serotonin in the whole blood (*F*_4,232_ = 0.56, *P* = 0.69) or for corticosterone concentrations (*F*_4,232_ = 1.74, *P* = 0.14).

## Discussion

Here, we present a comprehensive profile of feather pecking behavior in non-cage laying hens at the individual level. Approximately, half of the hens in this study were consistent in their pecking behavior, i.e., at least three out of five times, while the other half were not. Therefore, based upon their pecking behavior, half of the hens were classified as feather peckers at least once during their lifetime. These results further complicate the management of laying hens in large groups. Removing a bird that is observed to be feather pecking from the flock may be an ineffective management strategy since removed hens may or may not perform feather pecking again in the future. Therefore, once a feather pecker, always a feather pecker? Not necessarily.

Giving and receiving feather pecks are not mutually exclusive behaviors. Therefore, hens may have the appearance of a victim with the motivational drive of a feather pecker. Hens classified as PV appeared to perform more pecks than hens classified as P, yet they received comparable numbers of feather pecks as V hens. Therefore, these hens may have the phenotype of a V, but the motivation to peck as a P. Based on findings from previous research (31), hens are most likely to be engaged on both sides of a feather pecking interaction, so it was not wholly unexpected that most hens were classified as PV, or that they remained PV throughout the duration of the study.

Consideration should be made to the possibility that pecking behavior may have been missed due to the observational techniques utilized in this study. However, continuously observing hen behavior is impractical, so a conservative interpretation would suggest that the frequency of feather peckers and victims are a minimum, while the number of neutrals is at a maximum. Therefore, the proportion of the population engaged in feather pecking behavior may actually be underrepresented here.

The increasingly positive relationship between SFP and AP suggests that as hens age, they become established in their pecking behavior. Proactive copers have less behavioral plasticity than reactive copers, and this rigidity in behavioral patterns increases as hens age (32). It may be possible that the two different types of pecking may provide the giver with similar feelings, even though behaviorally they have different functions. An increase in GFP was observed in flocks of laying hens as they aged (5); however, the incidences of SFP and aggressive pecking did not occur often enough to discern a difference in their performance as hens aged. Alternatively, the hens performing these similar levels of SFP and AP may be becoming increasingly aggressive as they age. It can also be argued that SFP will usually be targeted at victims that are lower in the hierarchy, because otherwise the victim may respond aggressively to being pecked. This could result in an intermediate group of hens that shows relatively high levels of both aggressive pecking and SFP, directed mostly at individuals that are lower in rank.

It was not feasible to determine individual hen rank in this study; therefore, we were unable to identify whether social status impacted pecking behavior. With this in mind, consideration must be made to the impact of these results on commercially housed hens. The hens in this study were housed in small groups of 10 hens, a group size in which hierarchies are established via aggressive pecking. However, in larger groups of hens, no hierarchical structure has been observed and aggression levels are much lower (33). Hens housed in large groups have been observed to alter their aggression strategy so that they use their energy for immediate competitive interactions rather than establishing and maintaining a social hierarchy (34, 35). The relationship between SFP and AP strengthened as the hens aged. However, as feather pecking does not appear to be associated with aggression and that individual hen rank was not assessed in this study, it can be difficult to identify whether this relationship was due to maintenance of the social hierarchy or if the hens performing the SFP were more perseverative in all pecking behaviors and were therefore more likely to perform AP as well as SFP.

The patterns of feather pecking behavior observed in this group of hens appear to mimic the patterns of addictive behaviors in humans. Humans are variable in their propensity to develop and the severity of their addictions, which has been linked to differences in genetics, as well as neurological differences including pathological changes in neural circuitry involved in reward, motivation, cognitive control, and mood (36). Beyond their physical expression, many physiological parallels exist between these two detrimental behaviors.

Hens performing GFP are likely to escalate to performing SFP, and this escalation most often comes at the end of a GFP bout, suggesting that an SFP may signal a release or satisfaction of the pecking behavior for that moment. For addicts, a satiation high is attractive because it numbs the sensations of pain or distress that lasts until the sensation fades, causing the individual to re-engage in the addictive behavior (37). If feather pecking is a behavior that hens perform to cope with stress, perhaps hens become addicted to sensations generated by feather pecking. Both the tendency to develop feather pecking and the propensity for addiction are heritable (38); and similar to humans, individual hens may have different propensities for developing or breaking the addictive behavior (39).

Initiation and maintenance of addictions in humans, such as cigarette smoking can be influenced by affective state. For instance, some individuals are able to socially smoke [because it is a socially transmitted behavior (40)], with the ability to start and stop smoking without difficulty, while others wage a lifelong losing battle (those that continually smoke throughout their lifetime) due to multi-faced underlying motivating factors (41). Nicotine has been observed to release serotonin in the frontal cortex of rats (42) and antidepressants have been effectively used to aid smoking cessation in humans (43, 44). Female humans with an irritable temperament were more likely to begin smoking; and males with a depressive temperament were more likely to maintain the smoking behavior throughout their lifetime (45). Although addiction to smoking is partly induced by physical effects of nicotine, non-substance behavioral addictions show striking similarities with substance-related ones (46). Feather pecking is considered a socially transmittable behavior that varies in severity and perseverance, and has been linked to serotonergic sensitivity and brain morphology (47, 48). Therefore, if feather pecking behavior is described as an expression of anxiety and depression, then hens that are feather pecking may be performing addictive behaviors to find relief from their affective state – just as addicted cigarette smokers engage in this harmful behavior to feel better about a stressful situation.

Further supporting this idea, depressed cigarette smokers have lower brain serotonin function and higher lifetime aggression scores compared to non-smokers (49) – all factors characteristic of hens showing feather pecking behavior. The prevalence of feather pecking is impacted by genetics, while propensity to develop a smoking addiction differs by ethnicity and social economic class. Although cigarette smoking behavior can be altered through education and societal awareness, hens cannot benefit from the same type of approach to bring about cessation of feather pecking, emphasizing the importance of human caretakers’ responsibility to mitigate this behavior in laying hens.

Surprisingly, few physical measurements provided insight into the pecking behavior of hens. Feather scores were similar across behavioral categories, suggesting that feather cover may not be a reliable indicator for identifying victims of feather pecking, particularly if hens are beak trimmed as they may be less able to pull feathers out. Further, as corticosterone and serotonin levels were unchanged among the behavioral categories, peripheral measurements of the physiological response to stress may not be effective in identifying hens that are giving or receiving feather pecks.

This leaves the question of what makes the neutrals different. Tail biting in pigs (a compulsive behavior) follows very similar patterns of genotypic and phenotypic expression compared with feather pecking in laying hens. To this end, 19 genes exhibited different expression patterns from pigs that never engage (neutrals) in tail biting compared to pigs that are either performing or receiving tail biting (50). Yet pigs observed to engage in tail biting are not consistent in their biting behavior throughout the duration of their lifetime (51). Furthermore, since N hens had higher whole blood serotonin concentrations than PV or V at 21 weeks, this supports the theory that individuals that do not engage in feather pecking, either receiving or giving, may be different from the rest of the population. Previous research into individual hen behavior illustrates that extremely victimized hens do alter their behavior and movement in large groups of hens (52), so neutral hens may develop strategies to avoid becoming engaged in feather pecking events and warrants future exploration. Therefore, individuals having a neutral endophenotype may be less social and have a higher survivability because they are different from hens that perform or receive feather pecks. However, the number of individuals in this study who were consistently neutral was very small, creating opportunities to identify selection characteristics of hens who will consistently not engage in SFP behavior.

Importantly, the development of feather pecking is multifactorial and can be influenced by many environmental stimuli. Receiving feather pecks can be stimulated by feather cover and condition (53), so hens with poor feather cover may entice flock-mates to feather peck, even though the recipients are not behaving or look like a victim. Hens housed in bright lighting conditions (12), fed diets low in protein, minerals, or amino acids (54), in cool conditions, and drink from bell drinkers (55), or do not have access to litter (56) are likely to develop feather pecking behavior. Therefore, understanding the variation of feather pecking behavior in individuals is important, yet this information is only one piece of the puzzle. Just as patterns of cigarette smoking in the United States have changed throughout history due to social (40), economic (57), and political influences (58), feather pecking can manifest and subside for a variety of reasons.

The initiation of feather pecking behavior may stem from fear and anxiety, but the inability to mitigate this problem once it develops may be due to the addictive quality of this behavior. Therefore, not only do animal managers need to select for individuals that do not begin performing this behavior but also for hens that are less likely become addicted to the performance of feather pecking following a random feather pecking event or bout of normal GFP. Therefore, by selecting for hens that show a consistently neutral feather pecking behavioral phenotype as well as selecting against hens that are consistently feather peckers, we may be able to reduce the prevalence of feather pecking in laying hens, ultimately increasing their welfare state.

## Conflict of Interest Statement

The authors declare that the research was conducted in the absence of any commercial or financial relationships that could be construed as a potential conflict of interest.
